# Prediction of isometric forces from combined epidural spinal cord and neuromuscular electrical stimulation in the rat lower limb

**DOI:** 10.1038/s41598-024-66773-9

**Published:** 2024-07-09

**Authors:** Daniel Song, Matthew C. Tresch

**Affiliations:** 1 Department of Biomedical Engineering, Northwestern University, Chicago, IL 60611 USA; 2 Department of Physical Medicine and Rehabilitation, Northwestern University, Chicago, IL 60611 USA; 3 Shirley Ryan AbilityLab, Chicago, IL 60611 USA

**Keywords:** Spinal cord, Biomedical engineering

## Abstract

Although epidural spinal cord and muscle stimulation have each been separately used for restoration of movement after spinal cord injury, their combined use has not been widely explored. Using both approaches in combination could provide more flexible control compared to using either approach alone, but whether responses evoked from such combined stimulation can be easily predicted is unknown. We evaluate whether responses evoked by combined spinal and muscle stimulation can be predicted simply, as the linear summation of responses produced by each type of stimulation individually. Should this be true, it would simplify the prediction of co-stimulation responses and the development of control schemes for spinal cord injury rehabilitation. In healthy anesthetized rats, we measured hindlimb isometric forces in response to spinal and muscle stimulation. Force prediction errors were calculated as the difference between predicted and observed co-stimulation forces. We found that spinal and muscle co-stimulation could be closely predicted as the linear summation of the individual spinal and muscle responses and that the errors were relatively low. We discuss the implications of these results to the use of combined muscle and spinal stimulation for the restoration of movement following spinal cord injury.

## Introduction

Functional electrical stimulation (FES) is a useful approach for producing movements as well as facilitating recovery of voluntary movement after spinal cord injury (SCI)^1,2^. One type of FES, epidural spinal cord stimulation, has been used to evoke and/or modulate muscle activity to restore standing and stepping function after SCI^3–5^. Epidural spinal cord stimulation works mainly by recruitment of dorsal root afferents near the electrode site, which then activate motoneurons synaptically through recruitment of spinal networks^6,7^. In contrast, in another type of FES, stimulation is applied directly to motor axons or peripheral nerves to activate individual muscles^8^. This type of direct neuromuscular stimulation can be used to produce stepping patterns and other functional movements by many implanting multiple muscles across the limb and stimulating them with specific spatiotemporal patterns in order to produce the desired functional movements.^9–11^.

Both types of FES have strengths and weaknesses as approaches for restoring function after SCI. For instance, because epidural stimulation recruits spinal interneuronal networks, it is capable of evoking functional movements recruiting muscles across the limb, such as multijoint extension or flexion^12^. However, because the responses activate multiple muscles at the same time, it can be difficult to fine tune evoked movements according to specific task demands. Muscle stimulation, on the other hand, can activate muscles individually, thereby enabling more fine-tuned control of evoked movements to achieve task demands. However, implanting and controlling stimulation of many muscles to ensure coordinated and effective behavior is a challenging control problem, making this approach difficult to implement.

Combining spinal and muscle stimulation might overcome the shortcomings of each type of stimulation, enabling improved restoration of function following SCI compared to approaches using each type of stimulation separately. For example, epidural spinal cord stimulation might be used to evoke a whole limb extension movement for forward propulsion with additional stimulation of a knee extensor muscle to produce a more vertical thrust when walking up a slope. Similar modifications might be used to step over obstacles of different heights or change direction of locomotion.

In order for such a spinal and muscle co-stimulation approach to be useful, however, the responses from spinal and muscle stimulation should combine simply and predictably. There are many reasons that the responses evoked from combined spinal and muscle stimulation might be difficult to predict. For example, collision of action potentials^13^ produced by spinal and muscle stimulation of peripheral axons might result in smaller than expected responses. Another reason for nonlinear interactions between spinal and muscle stimulation could be synaptic integration of sensory inputs in the spinal cord; e.g. if spinal stimulation causes a subthreshold depolarization of motoneurons, adding proprioceptive inputs from muscle stimulation might cause those motoneurons to become suprathreshold and produce additional muscle activity. In such a case, responses evoked by combined stimulation would be larger than expected from a linear summation of responses evoked separately. By making the responses evoked from combined stimulation difficult to predict, such factors would increase the complexity of movement control using combined muscle and spinal stimulation.

On the other hand, there are reasons to expect that such nonlinear interactions between spinal and muscle stimulation would be minimal. Previous work using intraspinal stimulation showed that combined stimulation of two different sites in the spinal cord can usually be predicted by the linear vectorial summation of each site’s individual response^14–16^. However, there has been little work evaluating the linearity of combined spinal and muscle stimulation. In this study we evaluate this possibility, testing the hypothesis that co-stimulation of the spinal cord and a single muscle can be predicted by the linear summation of individual spinal and muscle responses.

## Results

### Spinal and muscle stimulation responses

**Figure 1:**
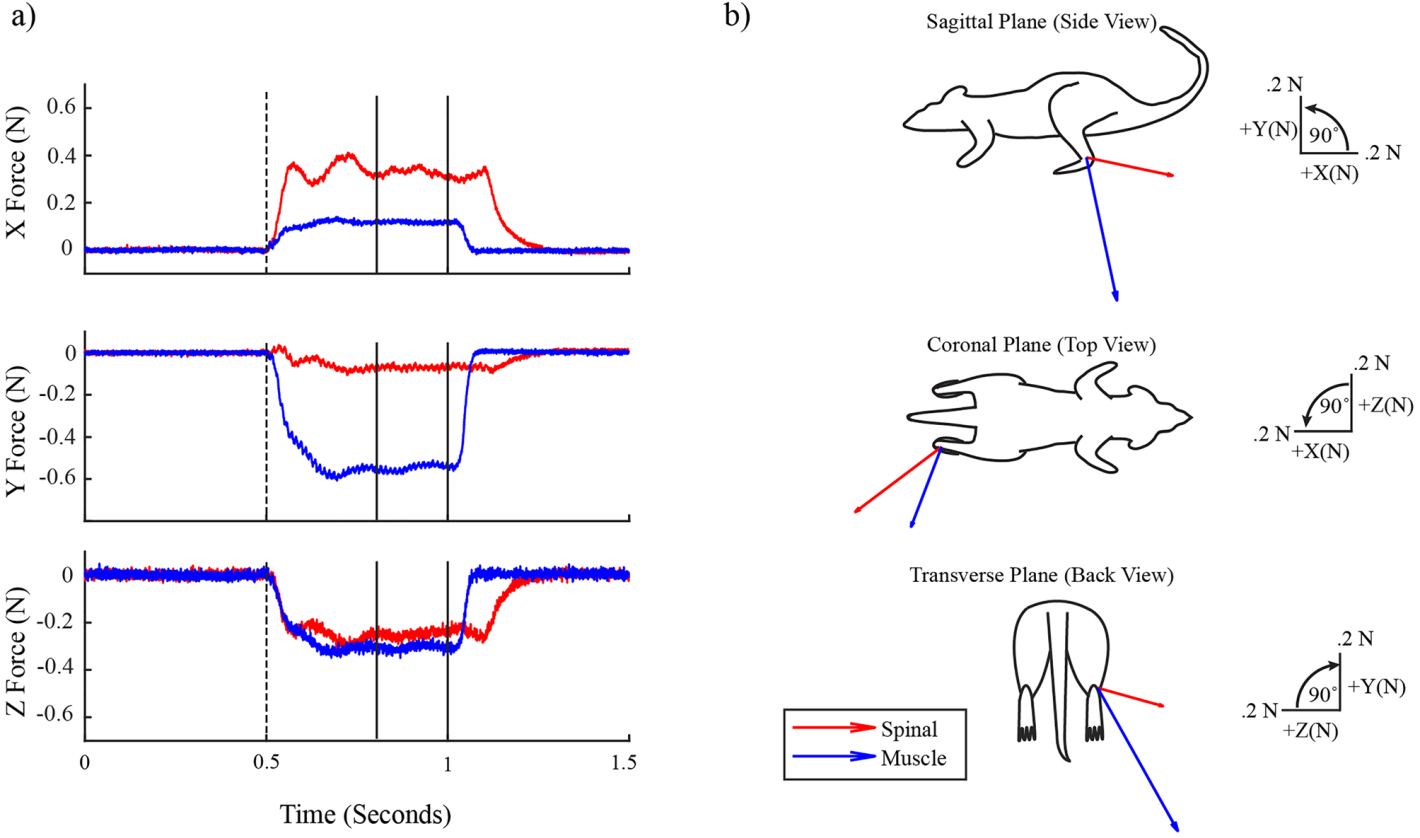
Example trials of stimulation delivered at VL and L5 spinal segment. (**a**) Force recordings in xyz dimensions in response to spinal (red) and muscle (blue) stimulation. Dotted vertical lines indicate the stimulation onset. Solid black lines indicate the time period used to calculate the isometric forces in subsequent analyses. (**b**) Corresponding isometric force vectors from a) in all three planes.

**Figure 2:**
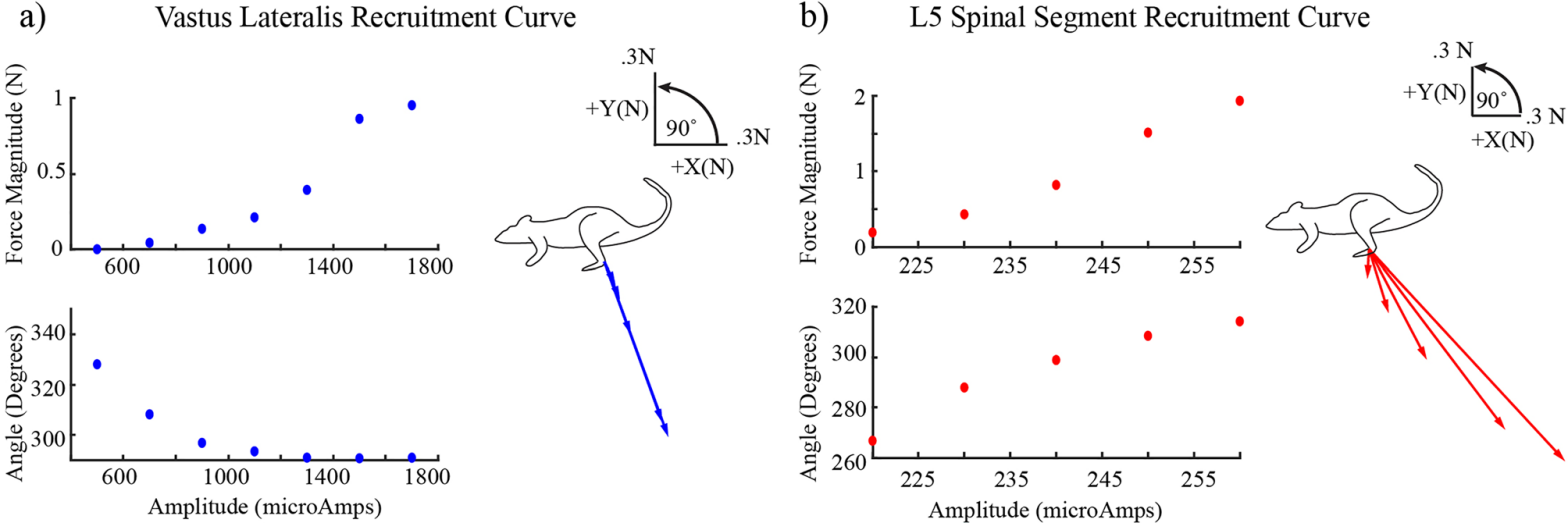
Example recruitment curves for stimulation at muscle and spinal sites illustrating how the magnitude (top) and direction (bottom) of the evoked responses change with stimulation strength. The corresponding vectors are shown on the right of each recruitment curve. (**a**) Recruitment curve in VL. (**b**) Recruitment curve in the L5 spinal segment.

Figure 1 shows an example of individual spinal and muscle stimulation trials for one animal. In this case, the forces evoked by spinal stimulation (red traces, Fig. 1a) showed initial transients early in the stimulation train before settling to a steady response, whereas forces evoked by muscle stimulation (blue traces) quickly reached a constant steady force. The steady portion of each force trace was used to find the isometric force vectors which were directed caudal and lateral for the spinal response and ventral and lateral for the muscle response (Fig. 1b). Figure 2 shows the recruitment curves from stimulation of the same muscle and spinal sites as in Fig. 1. Increasing the stimulation amplitude applied to the muscle caused a monotonic increase in evoked force magnitude with minimal change in force direction (Fig. 2a). Increasing the stimulation amplitude applied to the spinal site also caused a monotonic increase in evoked force magnitude, but the direction of the evoked forces changed as stimulation strength increased. Because of this change in direction with increasing stimulation strength, the range of force directions observed in individual spinal recruitment curves was considerably larger than those of muscle recruitment curves (sagittal plane angular deviation of 11.16° ± 11.23° for spinal vs. 1.38° ± 1.09° for muscle recruitment curves taken across n = 11 animals, Welch’s t test p = 0.01).

**Figure 3:**
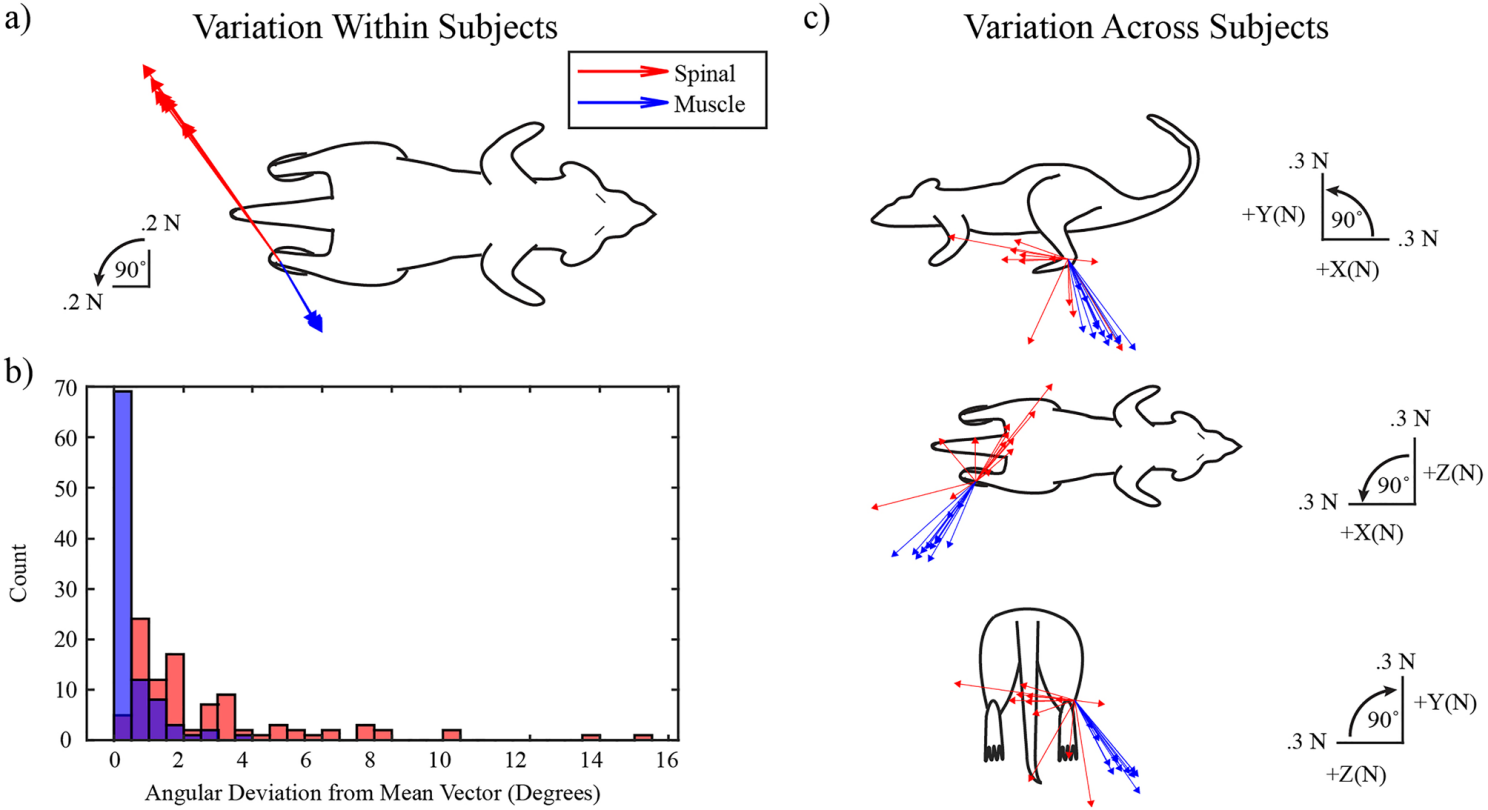
Characteristics of forces evoked by individual muscle and spinal stimulation. (**a**) Example of eight repeated spinal and muscle stimulation trials for a single subject. Force vectors were consistent over repetitions. (**b**) Histogram of within-subject angular deviations of spinal and muscle responses. Red indicates spinal trials and blue indicates muscle trials. Angular deviation was calculated by first finding the mean vector within each subject and then calculating the angle between that mean vector and individual trial vectors. Spinal angular deviations were larger than muscle angular deviations, but overall angular deviations remained relatively low (no more than 16 degrees). (**c**) Force vectors representing the mean magnitude and direction for each subject’s spinal or muscle stimulation response.

We further examined the characteristics of individual spinal and muscle stimulation responses at a single amplitude for each site. Stimulation amplitudes were selected to produce forces similar to ground reaction forces measured during rat locomotion and eight repetitions were performed for each subject. Within each subject, repeated stimulation of the L5 spinal segment was slightly more variable than repeated stimulation of the VL muscle (difference in angular deviation, Welch’s t test, p = 3.5 × 10^–11^). Nevertheless, individual stimulation trials for both spinal and muscle sites never deviated more than 16 degrees from the subject’s mean vector demonstrating that directions remained generally consistent across repetitions (Fig. 3a,b). We also examined the range of force directions evoked by spinal and muscle stimulation across subjects (Fig. 3c). Forces evoked by VL stimulation were consistently directed lateral and caudal, as expected for a knee extensor muscle at this configuration. Forces evoked by L5 spinal stimulation were generally directed medially but had a range of directions in the sagittal plane. (Fig. 3c).

### Evidence for linear summation of spinal/muscle co-stimulation

**Figure 4:**
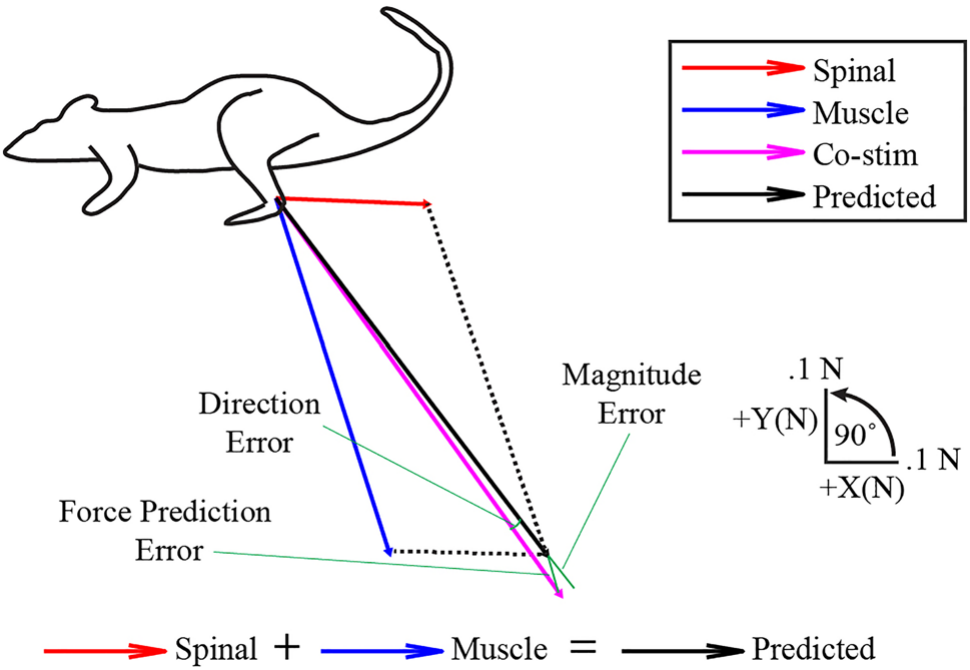
Example co-stimulation and prediction force vectors. The prediction vector was calculated by taking the linear summation of an individual spinal and muscle force vector collected within the same block of trials. Direct comparisons of co-stimulation and prediction vectors were made by calculating force prediction errors, magnitude errors and direction errors.

**Figure 5:**
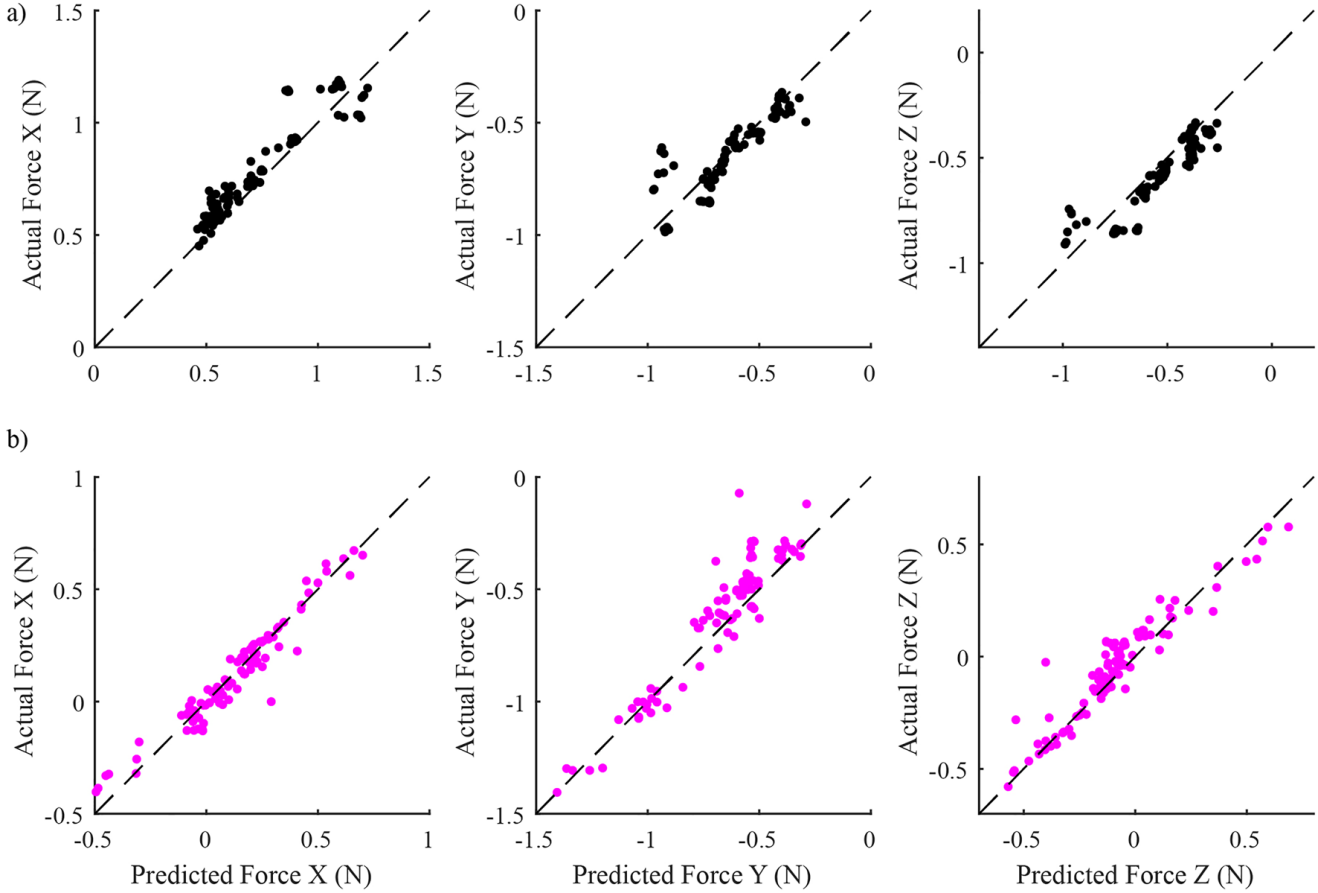
Linearity of co-stimulation assessed by combined R^2^ values which was calculated by taking the combined squared sum of residuals of each axis and dividing by the sum of all total sum of squares each axis (i.e. Each axis had its own total sum of squares). The dashed line indicates perfect prediction. (**a**) Linearity of muscle co-stimulation (VL and BF). The predicted and actual forces match closely for all axes (combined R^2^ = 0.75). (**b**) Spinal/muscle predicted forces also line up well with actual forces (combined R^2^ = 0.87).

Figure 4 shows an example of force vectors evoked by spinal, muscle, and combined spinal/muscle stimulation trials. When muscle and spinal stimulation were combined, the evoked response (magenta vector) was very similar to the response expected if the spinal and muscle combined linearly (black vector). We compared the linearity of combined spinal/muscle stimulation responses to that observed during muscle/muscle co-stimulation trials observed across all animals, since previous work has suggested that such muscle co-stimulation responses combine linearly^17^. A total of 96 muscle/muscle co-stimulation trials and 96 spinal/muscle co-stimulation trials were collected across twelve rats (8 muscle/muscle co-stimulation trials and 8 spinal/muscle co-stimulation trials per rat). The predicted co-stimulation forces were generally similar to the actual co-stimulation forces for both spinal/muscle and muscle/muscle trials (Fig. 5). The coefficient of determination with respect to unity (dashed line in Fig. 5) was 0.75 for muscle/muscle co-stimulation and 0.87 for spinal/muscle co-stimulation.

**Figure 6:**
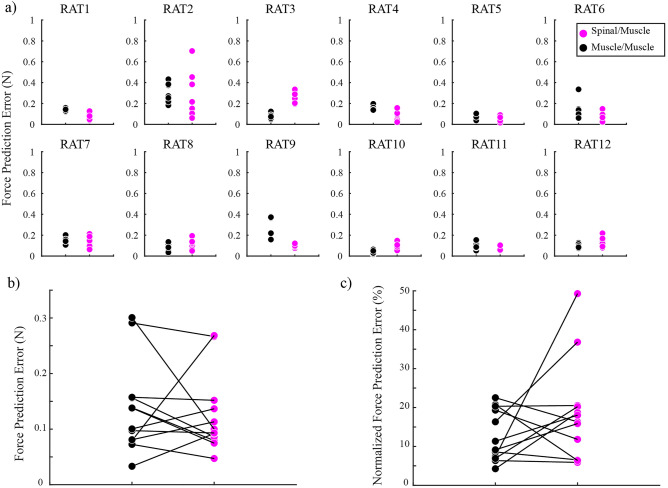
(**a**) Individual force prediction errors across all animals. Each dot represents the error observed on a single co-stimulation trial. (**b**) Comparison of mean force prediction errors. Each dot represents the mean force prediction error for each animal. (**c**) Similar data to (**b**) except force prediction errors are normalized to the predicted magnitude.

We next evaluated whether the deviations between predicted and observed co-stimulation vectors were greater for spinal/muscle as compared to muscle/muscle co-stimulation. We used muscle/muscle co-stimulation as a comparison to control for deviations from linearity due simply to force measurement or other experimental errors^16,17^. Force prediction error was calculated by taking the Euclidean distance between the observed co-stimulation force vector and the predicted co-stimulation force vector for each condition (see Fig. 3). Figure 6a shows the force prediction errors for muscle/muscle and spinal/muscle co-stimulation for each animal. The median force prediction error across all subjects and trials was 0.10 N for spinal/muscle co-stimulation (error normalized to predicted magnitude: 15%) and 0.12 N for muscle/muscle co-stimulation (error normalized to predicted magnitude: 10%). Using a linear mixed model with subject as random factor and co-stimulation type (spinal/muscle or muscle/muscle) as fixed factor, force prediction errors were not found to be significantly different for spinal/muscle cases compared to muscle/muscle cases (Fig. 6b). Similarly, neither direction error (p = 0.16) nor magnitude error (p = 0.63) was found to be significantly different when considered separately.

Previous work has shown that larger co-stimulation magnitude responses tend to be associated with larger errors^17^. Since predicted muscle/muscle co-stimulation magnitudes were on average larger than spinal/muscle magnitudes (muscle/muscle mean: 1.1 N vs. spinal/muscle mean: 0.76 N), it is possible that spinal/muscle errors might have appeared to be comparable to muscle/muscle errors simply because their magnitudes were smaller. We therefore repeated the linear mixed model including predicted co-stimulation force magnitude as a fixed effect, ‘nuisance’ variable. We found that the difference between spinal/muscle and muscle/muscle force prediction errors remained insignificant (p = 0.37) and there was no significant effect of the predicted magnitude (p = 0.27). The direction (p = 0.42) and magnitude errors (p = 0.38) also remained not significantly different after including predicted magnitude as a fixed effect. As another way to account for differences in magnitude, we also normalized the force prediction errors by the predicted magnitude, thereby expressing the errors as a percentage (Fig. 6c). A new mixed model using normalized force prediction error still showed that there was no significant difference (p = 0.28). Taken together, these results suggest that the apparent linearity of spinal/muscle responses was not due to magnitude differences.

**Figure 7:**
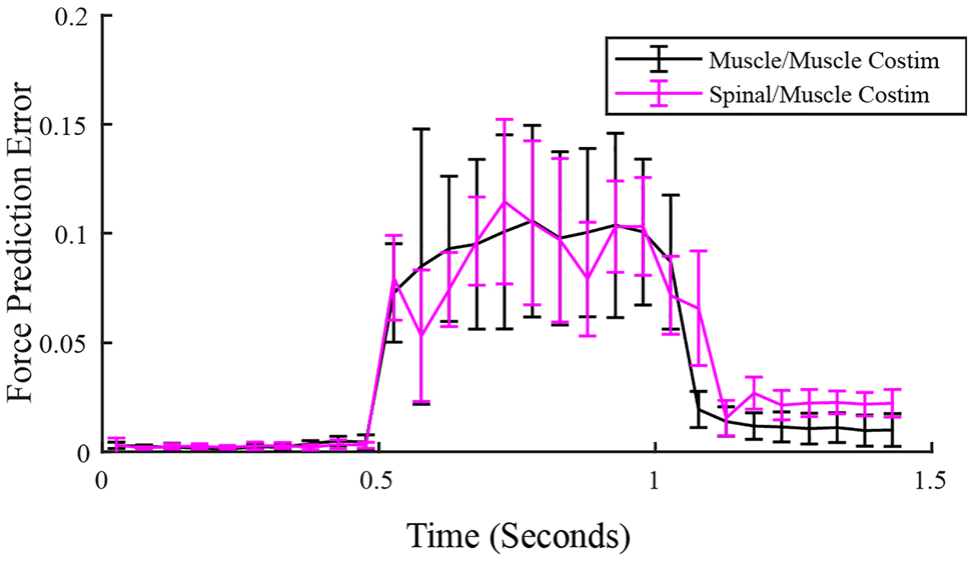
Force prediction errors across the stimulation duration for both spinal/muscle and muscle co-stimulation trials for a single rat. Each co-stimulation trial was separated into 50 ms time windows. A force prediction error was then calculated for each time window. Error bars represent the standard deviation of force prediction error at each window.

We also evaluated whether force prediction errors varied across the duration of the co-stimulation train, potentially reflecting temporal dynamics of spinal processing. Figure 7 shows the prediction errors for muscle/muscle and spinal/muscle co-stimulation at distinct time points in the stimulation train for a single animal. Across all animals, there was no obvious relationship between the size of the prediction error and when in the stimulation train the error was calculated. To evaluate this possibility, we compared prediction errors between early (time window: 100–300 ms) or late (time window: 300–500 ms) in the stimulation train, examining only spinal/muscle co-stimulation trials. We found no difference in error between these two periods of time in the stimulation train (p = 0.72).

## Discussion

We found that forces produced by spinal/muscle co-stimulation were generally similar to the responses predicted by the linear sum of the forces from separate stimulation of each site. The spinal/muscle co-stimulation prediction errors were not significantly different from muscle co-stimulation errors. These results suggest that spinal and muscle stimulation responses combine linearly and so combined muscle/spinal stimulation might be a useful approach for restoring function after SCI.

There were several reasons one might have expected nonlinear interactions between spinal and muscle responses. For example, collision of action potentials along motor or sensory axons might have led to smaller than expected responses from co-stimulation of spinal and muscle sites^13^. Alternatively, one might have expected evoked co-stimulation responses to be larger than expected due to synaptic integration within spinal circuits: activation of sensory afferents by muscle stimulation might cause subthreshold activation of spinal interneurons and motoneurons, which then become suprathreshold when muscle stimulation is combined with spinal stimulation. Similarly, recruitment of inhibitory pathways by spinal or muscle stimulation might have caused smaller than expected responses^18^. The linear combination of muscle and spinal responses observed here, on the other hand, is consistent with previous studies showing linear interactions between responses evoked by intraspinal stimulation^14–16^.

One explanation for the lack of nonlinear interactions observed here might be due to the fact that, although trains of stimulation were applied simultaneously to spinal and muscle sites, different frequencies of stimulation were applied to each site. Individual stimulation pulses to muscle and spinal sites were therefore not delivered at a consistent interval to one another. As a result, any interactions requiring precise and consistent pulse intervals (e.g. antidromic collisions, synaptic integration) might have been minimized in these results^19^. Future experiments in which the interval between spinal and muscle stimulation pulses are systematically varied will be required to examine this issue further.

Another explanation for the linearity observed here is that muscle and spinal stimulation might have recruited non-overlapping neural substrates. In the current study, we restricted our analysis to responses evoked by muscle stimulation of VL and spinal stimulation at L5 in order to ensure experimental consistency across animals. Given that VL motor pools are primarily located at more rostral spinal segments^20^, this choice of sites might have recruited minimally overlapping spinal substrates and predisposed our study to find predominantly linear interactions between muscle and spinal responses. However, responses evoked by spinal stimulation often involved large movements of the entire limb, suggesting broad recruitment of motor neurons across the spinal cord which might have allowed for observing nonlinear interactions if they were present. In this context, it is interesting to note that although there were no consistent nonlinear interactions between spinal and muscle stimulation across animals, some individual animals showed larger predictions errors for spinal/muscle co-stimulation trials as compared to muscle/muscle trials (see RAT3 in Fig. 6). It might be that slight variations in the position of the spinal stimulation electrode or in the anatomy of spinal roots in this animal recruited different motor pools as compared to other animals^21^, potentially resulting in the observed nonlinear interaction. Future experiments recording electromyograms across muscles while stimulating different spinal sites might evaluate this possibility more directly.

Spinal stimulation can also be sensitive to time-varying descending neuromodulation influencing the intensity of evoked responses. For instance, monoaminergic projections from the brainstem can enhance the gain of motor units, greatly increasing their response to synaptic inputs^22,23^. Such modulatory effects might have been expected to decrease the predictability of spinal responses or resulted in non-linear interactions. However, we collected data points close in time (45 s between trials) to minimize any variations in the modulatory state of spinal systems over time. Additionally, these experiments were performed under anesthesia which might have reduced such modulatory effects. Future experiments examining combined stimulation of different spinal and muscle sites in unanesthetized animals with SCI will be required to evaluate these issues more thoroughly.

Whether these results in rats would also be observed in humans remains unclear, especially given the differences in spinal cord sizes and anatomy between species^21^. For example, stimulation at the L5 spinal segment in humans might recruit activity in a different set of muscles than those observed in this study and so evoked responses might interact with muscle stimulation differently. Additionally, to standardize spinal stimulation, we used the same parameters across all animals. In contrast, clinical applications of epidural spinal cord stimulation in humans typically identify a unique set of stimulation parameters for each patient to achieve tasks like standing or stepping^24,25^. Although this specialized tuning might produce the same desired outcome in all patients, using different parameters might recruit different spinal circuits between patients. This approach might thereby potentially affect the chance of nonlinear interactions with responses evoked by muscle stimulation. Additional experiments could incorporate electromyograms at multiple muscles to identify which muscles are recruited and whether nonlinear interactions occur between spinal and muscle stimulation.

We also observed that the direction of responses evoked by stimulation of an epidural spinal site varied as stimulation amplitude was increased. This result might simply reflect recruitment of additional axons near the electrode with increasing stimulation strength. This result does potentially complicate any control scheme using spinal stimulation for the precise restoration of movement, since movement magnitude cannot be easily controlled independently of movement direction although we note that the change in evoked force directions was relatively small (less than 12 degrees on average).

The observed linearity between combined muscle and spinal stimulation, if found to be robust across species and stimulation conditions, might be exploited in applications to restore movement following spinal cord injury. The fact that the response resulting from combined muscle and spinal stimulation can be predicted as a linear combination of their separate responses greatly simplifies this type of control scheme.

## Methods

### Ethical approvals

All procedures were approved by the Institutional Animal Care and Use committee at Northwestern University and performed in accordance with the relevant guidelines and regulations. All procedures are also in accordance with the essential 10 ARRIVE guidelines^26^.

### Surgical procedure

A total of twelve female Sprague–Dawley rats (250–350 g) were used in this study. Rats were anesthetized with an intraperitoneal injection of urethane (1.5 mg/kg). Urethane was used due to its relatively minimal effect on reflex responses as compared to other anesthetics^27^. The animal’s level of anesthesia was monitored by the toe pinch response, respiratory rate, and whisker movements. Supplemental doses were administered as needed to achieve a deep anesthetic plane. The animal’s temperature was monitored using a rectal thermometer. Temperatures were kept between 36 and 38 °C using a heating pad controlled by a DC temperature controller (40-90-8D, FHC Inc.).

A laminectomy was then performed at the L1 vertebra to expose spinal segments L4 through L6^28^. The exposed spinal cord was then covered and kept moist with a cotton ball soaked in saline for the remainder of the surgery. Incisions were then made to expose the illium and ischium contralateral to the stimulation site. A small hole was drilled into each site and threaded rods (IMEX Veterinary Inc.) were then screwed into the holes.

After the muscle electrodes were implanted, the animal was then transferred to a raised platform. The pelvic posts were secured using an articulated holder (Noga Engineering). Lateral portions of L2 and L1 vertebra were dissected away to expose the transverse processes. Two clamps on either side of the spinal column were then used to stabilize the spinal column during stimulation. The right ankle was wrapped in a cuff and attached to a 6-axis force transducer (ATI-mini 40, ATI Industrial Automation Inc.). The force transducer was positioned such that the animal’s knee and hip joint were both approximately 90°. The clamps were tightened to minimize skeletal movements and ensure forces were isometric. Upon completion of the experiment, animals were euthanized by intraperitoneal injection of Euthasol followed by a thoracotomy.

### Electrodes and implantation

Spinal stimulation was performed with a Teflon coated 203 µm diameter solid silver electrode (786500, AM Systems Inc.). The stimulation electrode was initially positioned at the rostral border of the intact L2 vertebra. It was then moved 2 mm rostral and 500 µm laterally to the right. Dissections performed after the experiment showed that this approximately positioned the electrode on the caudal portion of the L5 entry zone. Correct placement of the electrode was verified by observing right hindlimb movements in response to single pulse stimulation. The spinal cord was then bathed in mineral oil.

Muscle stimulation was performed using custom-made electrodes. Teflon coated multi-stranded wire (793200, A-M systems Inc.) was stripped to expose a 1 mm section of bare wire. To increase surface area and reduce current density, a stainless steel tube (23R304-1 mm, Ziggy’s Tubes and Wires) was crimped over the exposed wire which was then filled with silver epoxy that was left to cure over several days. This resulted in a contact area of roughly 1 mm × 1 mm. For implantation, an incision was made over the hindlimb parallel to the femur to expose the vastus lateralis (VL) and biceps femoris posterior (BF) muscles. Motor points were found by probing various locations on the surface of the muscle that generated the most complete visual muscle contraction in response to a 1 Hz suprathreshold stimulation using the electrode contact. The electrode contact was then threaded through the muscle near the motor point using a hook needle. Return electrodes were made by exposing several millimeters of bare wire and were placed subcutaneously on each side of the belly.

### Stimulation

Biphasic, charge balanced stimulation trains were delivered with an isolated battery powered stimulator (SI-8, Tucker Davis Technologies Inc.) to evoke hindlimb isometric contractions. A digital bioamp processor (RZ5, Tucker Davis Technologies Inc.) was used to synchronize force transducer data with stimulation timing. Spinal stimulation parameters were 15 ms period, 0.2 ms pulse width and 600 ms train duration (40 pulses). Similar parameters were used to control hindlimb forces with spinal stimulation in previous work^29^. Muscle stimulation parameters were 13 ms period, 0.12 ms pulse width and 520 ms train duration (40 pulses). These parameters provided fused tetanic contractions^17,30^.

A range of amplitudes was selected to identify the range of forces produced by each stimulation site (L5 spinal segment, VL and BF) and characterize recruitment curves. For each trial, the average baseline forces 400 ms prior to stimulation were subtracted to demean the force traces. Isometric forces were calculated using the average force in a 200 ms window beginning 300 ms after stimulation onset. After the recruitment curve was generated, a single amplitude was selected that produced a force representing typical ground reaction forces observed during rodent locomotion. Ground reaction forces during stance phase typically range from 0.5 to 1.8 N for a rat weighing 275 g^31^. The mean spinal magnitude (0.55 N ± 0.27 N) and the mean muscle magnitude (0.7 N ± 0.21 N) both fell within this range and were therefore representative of forces that might be observed during locomotion. Stimulation trials were then performed in blocks. One block included a spinal only trial, muscle only trial and co-stimulation trial. The order of the trials in a block was randomized and blocks were repeated 8 times. Each trial was separated by at least 45 s of rest.

### Linearity analysis

Analyses were performed using custom scripts in MATLAB Version 2022a (Mathworks, Natick, MA). Circular statistics were performed using the CircStat Toolbox^32^. The prediction vector was calculated from the sum of the vectors evoked from stimulation of each site individually, using the spinal and muscle responses collected within the same block as the co-stimulation trial. Force prediction errors were calculated as the Euclidean distance between the prediction vector and observed co-stimulation vector. Magnitude errors were calculated as the absolute difference between the magnitudes of the observed and predicted co-stimulation vectors. Direction errors were calculated as the angle between predicted and observed vectors (Fig. 3). Normalized force prediction errors were calculated by dividing raw force prediction errors by the predicted force magnitude.

We compared the errors found by co-stimulation of an epidural spinal site and a muscle site to the errors found by co-stimulation of two different muscles (VL, BF) within each animal. Responses evoked from stimulation of two muscles have been observed to combine linearly, especially for muscles well separated in the limb such as VL and BF used here^17^. Because repeated data points within each animal are correlated, we could not pool all data across animals and use a t test or ANOVA which assumes data points are independent from one another. We therefore used linear mixed models to assess whether errors differed significantly between the two types of co-stimulation trials. The variation between subjects and trial-to-trial variability within each subject was accounted for by including subject as a random factor allowing for subject slopes and intercepts to be random effects. Subject intercept and slopes were also allowed to be correlated. Co-stimulation type (spinal/muscle vs muscle/muscle) was set as a fixed factor. Separate mixed models were fitted for each of the four errors (force prediction, normalized force prediction, magnitude, and direction). Errors were log transformed to satisfy the model’s assumption of normal distribution of residuals and homoscedasticity. In subsequent analyses, the predicted force magnitude evoked by co-stimulation was also included as a fixed factor since larger co-stimulation force magnitudes could potentially correlate with larger force prediction errors^17^. A maximum log likelihood estimator was used to fit model parameters.

### Sample size

We performed a sample size estimation by simulating force prediction errors based on the statistical model used in our analysis. Data points were simulated using a predefined covariance matrix for normally distributed model parameters and repeated 1000 times. We determined a sample size of 12 with eight data points per subject was adequately powered (80%) to detect a 0.1 N difference in errors between muscle/muscle and spinal/muscle co-stimulation with a significance threshold of 0.05.

## Data Availability

Datasets generated in this study are available from the authors upon reasonable request.
